# CD2 Immunobiology

**DOI:** 10.3389/fimmu.2020.01090

**Published:** 2020-06-09

**Authors:** Christian Binder, Filip Cvetkovski, Felix Sellberg, Stefan Berg, Horacio Paternina Visbal, David H. Sachs, Erik Berglund, David Berglund

**Affiliations:** ^1^Department of Immunology, Genetics and Pathology, Section of Clinical Immunology, Uppsala University, Uppsala, Sweden; ^2^Research and Development, ITB-Med AB, Stockholm, Sweden; ^3^Department of Medicine, Columbia Center for Translational Immunology, Columbia University Medical Center, New York, NY, United States; ^4^Division of Transplantation Surgery, CLINTEC, Karolinska Institute, and Department of Transplantation Surgery, Karolinska University Hospital, Stockholm, Sweden

**Keywords:** CD2, CD58, LFA-3 (lymphocyte functional antigen-3), T cell activation, costimulation, costimulation blockade, Alefacept, siplizumab

## Abstract

The glycoprotein CD2 is a costimulatory receptor expressed mainly on T and NK cells that binds to LFA3, a cell surface protein expressed on e.g., antigen-presenting cells. CD2 has an important role in the formation and organization of the immunological synapse that is formed between T cells and antigen-presenting cells upon cell-cell conjugation and associated intracellular signaling. CD2 expression is upregulated on memory T cells as well as activated T cells and plays an important role in activation of memory T cells despite the coexistence of several other costimulatory pathways. Anti-CD2 monoclonal antibodies have been shown to induce immune modulatory effects *in vitro* and clinical studies have proven the safety and efficacy of CD2-targeting biologics. Investigators have highlighted that the lack of attention to the CD2/LFA3 costimulatory pathway is a *missed opportunity*. Overall, CD2 is an attractive target for monoclonal antibodies intended for treatment of pathologies characterized by undesired T cell activation and offers an avenue to more selectively target memory T cells while favoring immune regulation.

## Introduction

Costimulation is needed in addition to antigen-specific signaling through the T cell receptor CD3 complex (TCR/CD3) to achieve full T cell activation. Stimulation of T cells through TCR/CD3 in the absence of costimulation has been shown to induce anergy (1–3). An additional way to induce T cell activation and differentiation is via cytokines that bind corresponding receptors on the surface of T cells (4, 5). Recent decades have seen the development of biologics which target costimulatory cell surface molecules and thereby induce immune modulatory effects (6–8). Interestingly, blockade of a single costimulatory pathway with these biologics induced immune modulatory effects despite multiple costimulatory pathways working in tandem during T cell co-stimulation (9). This suggests that certain costimulatory pathways may have unique functions which cannot be compensated for by other pathways involved in co-stimulation. Therefore, blockade of individual costimulatory pathways has the potential to induce unique immune modulatory effects and intentionally direct T cell activation toward a desired outcome.

CD2 is a transmembrane glycoprotein of the immunoglobulin superfamily expressed on the surface of T cells, NK cells, thymocytes and dendritic cells (10, 11). The corresponding binding partner of CD2 is lymphocyte-associated antigen 3 (LFA3; also known as CD58) which is expressed on the surface of B cells, T cells, monocytes, granulocytes, thymic epithelial cells (10, 12). In addition, CD2 also binds to CD48, albeit with a relatively lower affinity (13). Although CD2 has been known for several decades to participate in a costimulatory pathway of T cell activation, studies of other costimulatory pathways with effects of greater magnitude in mice, have, until now, received considerably more attention by immunologists.

The CD2 antigen was identified as one of the earliest T cell markers (14, 15) and was shown to be important for thymocyte development (16). However, research was complicated by the fact that CD2 may not have the same role in murine immunity as in the immune system of higher animals. Notably, only a minor percentage of B cells express CD2 in humans (17) while CD2 is broadly expressed on murine B cells (18). Further, mice lack expression of LFA3 which is the main binding partner of CD2 in humans. Mice express CD48, however CD48 has a relatively lower affinity for CD2 and can bind to both CD2 and CD244 (13). Thus, the degree to which data about the role of CD2 in murine immunity can be extrapolated to a human context is uncertain and must be practiced cautiously. This difference made development of suitable animal models for research of CD2-targeting therapies resource-intensive as only transgenic rodents and primates were relevant pre-clinical models. Consequently, most research about CD2 immunobiology was conducted *in vitro*. However, the high degree of conservation of CD2 in rodents and higher mammals suggests an important role in all organisms as conservation without selective pressure is highly unlikely (19).

Recent clinical studies have shown the importance of this pathway for in T cell activation, especially in the transplantation setting. We have therefore considered it appropriate and timely to undertake the present review of the CD2 immunobiology, overlooking of which would represent a lost opportunity (9). Further, we consider potential therapeutic applications of CD2-targeting biologics.

## Structure of CD2

**Figure 1:**
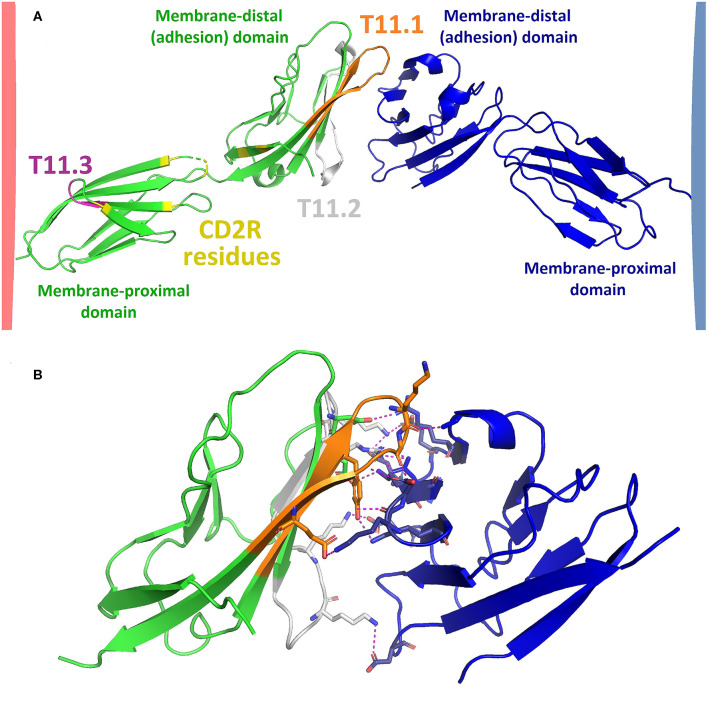
**(A)** Model of CD2 (green; Protein data bank code: PDB 1HNF) binding to LFA3 (blue; PDB 1CCZ). Model was created by structural alignment of PDB 1HNF [2.5 Å; (22)] and PDB 1CCZ [1.8 Å; (23)] with an X-ray crystallography structure of the adhesion domains of CD2 and LFA3 in their bound state [3.2 Å; PDB 1QA9; (24)] using PyMOL^TM^ 2.3.2 software (Schrodinger LLC, New York). The extracellular domain (ECD) of CD2 consists of a membrane-distal and a membrane-proximal Ig domain which are connected by a flexible linker region. The membrane-proximal region is linked to a transmembrane helix while the membrane-distal domain binds to LFA3. The most well-characterized CD2 epitopes are T11.1 (orange), T11.2 (gray) and T11.3 (purple), as identified by Peterson and Seed (25) and Damschroder et al. (26). CD2 undergoes a conformational change upon T cell activation and/or LFA3 binding which exposes an epitope called CD2R. CD2R residues as identified by Li et al. (27) are shown in yellow. The distance between T cell and APC upon CD2-LFA3 association according to this model would be approximately 130 Ångström. **(B)** CD2 (green) and LFA3 (blue) adhesion domains (PDB 1QA9) mainly associate via polar interactions (dotted yellow lines) such as hydrogen bonds and salt bridges. Amino acids involved in polar interactions between CD2 and LFA3 are shown as sticks, the remainders of both chains are shown in ribbon representation.

Human CD2 consists of 327 amino acids (351 amino acids including signal sequence) and a protein weight of ~40 kDa. References to specific amino acids throughout this paper refer to the position in the sequence of human CD2 including the signal peptide [Uniprot ID P06729; (20)]. Fully-glycosylated CD2 can weigh up to 50 kDa (21). The extracellular domain (ECD) of CD2 (amino acids 25–209) consists of two Ig domains, as shown in Figure 1A (22), and is linked to a disordered proline-rich cytoplasmic tail via a transmembrane helix. The membrane-proximal domain of the CD2 ECD connects the adhesion (membrane-distal) domain to the cell membrane and may be involved in CD2 clustering following T cell activation (27, 28). The membrane-distal domain is responsible for LFA3 binding. The binding surface consists of a flat beta sheet with relatively poor surface complementarity to the adhesion domain of LFA3. Interaction of CD2 with LFA3 is mainly mediated by polar interactions, as illustrated in Figure 1B. The ECD of CD2 has three glycosylation sites (N89, N141, and N150) with one being situated in the membrane-distal domain (N89) and two in the membrane-proximal domain [N141 and N151; (29)]. In folded CD2, N89 is situated near several amino acids which are important for siplizumab binding [N42, K79, and T83; (26)] thus the N89-linked glycan may be part of siplizumab's binding site.

There exist three well-characterized CD2 epitopes: T11.1, T11.2, and T11.3 (25, 30). T11.1 and T11.2 are located in the adhesion domain and are involved in LFA3 binding (16). T11.3 is part of the membrane-proximal domain (25) and exposure of this epitope increases upon T cell activation or CD2 clustering (27). T11.3 overlaps with another CD2 epitope named CD2R. The name of CD2R derives from its exposure being restricted to certain conformational states of CD2. While the T11.3 residues identified by Peterson and Seed (25) are not identical to the residues of CD2R as identified by Li et al. (27), both are located in the same region of CD2, namely in the membrane-proximal domain and the flexible linker region. The narrow distance between cells created by CD2-LFA3 as modeled in Figure 1A is approximately 130 Å, similar to the distance created by TCR bound to peptide-loaded major histocompatibility complex (TCR-pMHC) and CD4-MHC (31). This is essential for the kinetic segregation of activating and inhibiting membrane molecules upon formation of the immunological synapse [IS; (32)]. Presumably, siplizumab binds to T11.2 and T11.3 while Alefacept binds to T11.1 and T11.2 like LFA3. Alefacept, but not siplizumab, compete for CD2 binding with LFA3 (26).

Characteristic of cell-cell adhesion proteins, CD2 and LFA3 interact with relatively low affinity for each other (K_d_ = 9–22 μM) and a fast dissociation rate (33). Similar observations have been made for CD28/CD80 [K_d_ = 4 μM; (34)], LFA-1/ICAM-1 [K_d_ = 0.5 μM; (35)], CD2/CD48 [K_d_ = 100 μM; (36)] and CD40/CD40L [K_d_ = 0.2 μM; (37)]. A relatively low affinity and a high dissociation rate may facilitate cellular scanning but in the absence of further specific interactions of e.g., TCR with p-MHC (T cells) or of CD16 with an Fc-fragment (NK cells) no stable IS will be formed. Even though costimulatory molecules have a relatively low affinity for each other, association of thousands of costimulatory membrane molecules may still result in considerable cell-cell avidity. Furthermore, both CD2/LFA3 affinity and LFA1/ICAM1 affinity increase following cellular activation, providing another mechanism by which cell-cell avidity can be increased upon specific cell-cell conjugation (38–40).

**Figure 2:**
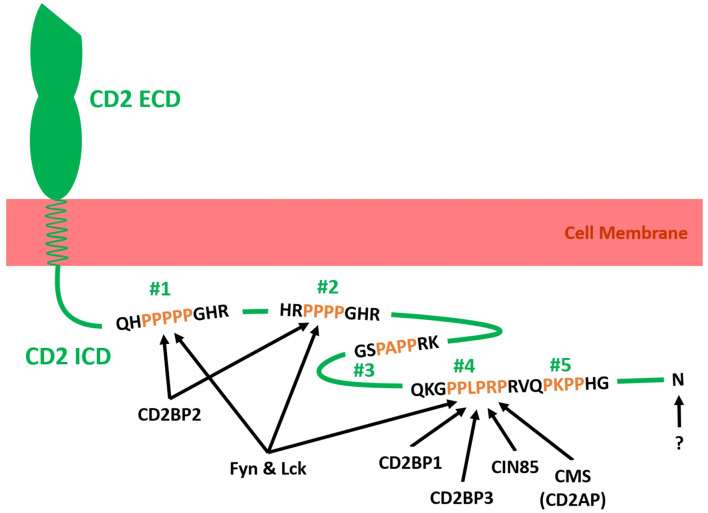
Schematic illustration of adaptor molecules binding to the intracellular tail domain (ICD) of human CD2 (Green). The ICD of CD2 is connected to the extracellular domain (ECD) of CD2 via a transmembrane helix in the cell membrane (red). The ICD of CD2 contains five SH3 binding domains (Two proline residues separated by two or three amino acids and flanked by a basic amino acid residue; #1–#5), of which two can also act as GYF-binding motifs (#1 and #2). Fyn kinase and CD2-binding protein 2 (CD2BP2) have been shown to bind to #1 and #2 (41). Additionally, Fyn kinase binds to #4 and/or #5 (42). Lck kinase has been shown to interact with #1, #2 and #4 in rat CD2 ICD (43). CD2BP1, CD2BP3, Cbl-interacting protein of 85 kDa (CIN85) and Cas ligand with multiple SH3 domains (CMS; Human form of CD2-associated protein CD2AP) bind to #4 (44, 45). The C-terminal amino acid N327 has been shown to be essential for CD2-LFA3 affinity regulation but no binding partner has been identified (38, 46). The ICD of CD2 precipitated with the T cell receptor complex (TCR/CD3) and Phosphatidylinositol-3 kinase (PI3K) but no direct interaction and binding motif has been identified (47, 48). Further direct interactions between the cytoplasmic tail of CD2 and intracellular or membrane proteins may exist but have yet to be identified.

The proline-rich intracellular domain (ICD) of CD2 is highly conserved across mammalian species. Considering how much time has passed since their divergence from a common ancestor, this degree of conservation indicates that CD2 fulfills an important function in mammalian immunity (19). As illustrated in Figure 2, the intracellular tail of human CD2 contains five potential src homology 3 domain (SH3)-binding sites [PxxP or PxxxP; (49, 50)], of which the first and second can also function as glycine-tyrosine-phenylalanine (GYF)-binding sites [PPPPGHR; (41, 51)]. The cytoplasmic tail of CD2 has been shown to interact with fyn kinase and lck kinase (41–43), CD2-binding protein 1 [CD2BP1; (52)], CD2BP2 (41, 49, 51), Cbl-interacting protein of 85 kDa [CIN85; (45, 53)], CD2BP3 [Variant of CIN85; (52)], and Cas ligand with multiple SH3 domains (CMS, also called CD2 adaptor protein or CD2AP). Further, while no direct interaction has been proven, CD2 has been reported to precipitate with TCR/CD3 and PI3K (47, 48).

## Role of CD2 in Actin Cytoskeleton Rearrangement And Cellular Signaling

Intracellularly, CD2 influences rearrangement of the actin cytoskeleton and agonistic cell signaling. These two functions seem to be spatially separated as a fraction of CD2 molecules transitions to lipid rafts in the cell membrane upon cell activation where it co-localizes with other signaling molecules important in transducing TCR/CD3- and costimulatory receptor-mediated signaling, e.g., activated protein tyrosine kinases (41, 54, 55). In contrast, molecules associated with connecting CD2 and the actin cytoskeleton remain outside of lipid rafts (41, 52).

Lateral mobility of CD2 in the plasma membrane of T cells has been shown to decrease following activation (39, 56, 57). Immobilization of CD2 is likely caused by crosslinking of the cytoplasmic tail with the actin cytoskeleton (56). As depicted in Figure 2, CMS, CIN85, and CD2BP3 compete for binding to the fourth SH3-binding domain in the cytoplasmic tail of CD2 (52). CMS and CIN85 connect CD2 to the actin cytoskeleton in several ways. Both have been shown to associate with the actin capping protein capZ (45, 58) and adaptor protein p130Cas (53, 59). Further, CMS was reported to associate with the actin regulatory protein cortactin (60) and directly with F-actin (61). Interestingly, Tibaldi and Reinherz (52) showed that degradation of CIN85 and CD2BP3 increases following T cell activation and that CIN85 inhibits cell polarization while CD2BP3 does not seem to influence cell polarization. Given that CMS binding to CD2 seems activation-dependent (42), a model was proposed where CIN85 and CD2BP3 occupy SH3-binding domain 4 in the cytoplasmic tail of CD2 in resting T cells while their degradation frees up this site for binding by CMS upon cellular activation. Consequently, reorganization of the actin cytoskeleton and polarization of the microtubule organizing center (MTOC) toward the IS can occur. Indeed, CD2 and CMS facilitate polarization of the actin cytoskeleton and T cells expressing truncated CD2 mutants lacking the C-terminal 20 amino acids of the cytoplasmic tail do not display polarization toward the IS (42, 62). Furthermore, CD2BP1 also binds SH3-binding domain 4 and locates to the IS upon cell activation where it links CD2 or CMS and Wiskott-Aldrich syndrome protein (WASp) to facilitate actin cytoskeleton rearrangement (54).

The TCR/CD3 complex, particularly the CD3 zeta chain is involved in transducing CD2-mediated activation signaling (28, 63–66). However, no direct association between CD2 and TCR/CD3 has been proven, even though they have been reported to co-immunoprecipitate (47). Considering the evidence discussed above, co-localization of CD2 and TCR/CD3 may, at least in part, be mediated through adaptor proteins and the actin cytoskeleton. Future research should investigate the interaction of CD2 and the actin cytoskeleton through adaptor proteins more closely, as it seems to play an important role in IS formation (28) and transport of lytic granules [CTLs; (62)]. Another unanswered question is whether CD2 similarly influences transport of secretory cytokines.

The network of CD2-mediated activation signaling overlaps partly with TCR/CD3-mediated signaling and is dependent on protein tyrosine kinases like lck and fyn (55, 67, 68, 70). Indeed, at least part of CD2-mediated signaling seems to depend on CD3ζ expression (28, 65, 66). Similar to TCR/CD3, CD2 elicits activation signaling through the lck/CD3ζ/ZAP70/LAT/SLP76/Ras-MAPK pathway (28, 43, 69, 70). Additionally, CD2 can induce activation signaling via the PLCγ1/calmodulin/calcineurin/NFAT and PLCγ1/Vav1/PKCθ/Dok/FAK/Pyk2/JNK axes (57, 68, 71). Preliminary evidence analyzing the kinome of CD2-mediated signaling in CTLs confirmed these findings and elucidated a broad network of CD2-induced signaling which influences IS assembly, cell polarization, T cell activation and metabolic regulation which overlapped only partially with known TCR/CD3- or CD28-mediated signaling pathways (62). Generally, CD2 costimulation has been shown to act additively with TCR/CD3- and CD28-mediated signaling and to induce strong mTOR signaling but weak NF-κB activity when compared to CD28 costimulation (62, 69, 71, 72). While the majority of the literature investigating CD2 signaling has been conducted in T cells, there are indications that CD2-mediated signaling in NK cells utilizes the same pathways as T cells (73–75).

The role of CD2BP2 in CD2-mediated signaling is largely unexplored. CD2BP2 binds proline rich regions one and two in the cytoplasmic tail of CD2. Evidence surrounding the role of CD2BP2 in CD2-mediated signaling suggests that it specifically enhances CD2-mediated activation signaling but not TCR/CD3-mediated signaling (36). The nature of how CD2BP2 acts in intracellular signaling is poorly.

Even though costimulatory signaling pathways were originally assumed to be largely redundant, there are differences in the quantity and quality of signaling that is elicited. Specifically, blockade of CD2/LFA3 co-stimulation may have distinct effects on cell metabolism and activation distinct from those observed in e.g., CD28/B7 co-stimulatory blockade.

## The Role of CD2 in the Immunological Synapse (IS)

**Figure 3:**
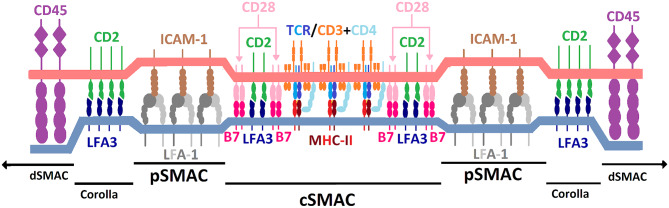
Schematic illustration of the immunological synapse and spatial distribution of TCR/MHC and costimulatory molecules. Illustration is for explanatory purposes and relative sizes of different molecules are not necessarily to scale. Regions include central supramolecular activation cluster (cSMAC), peripheral SMAC (pSMAC), CD2/LFA3 corolla and distal SMAC (dSMAC). CD2 (green) is positioned in the T cell plasma membrane (light red) and locates to both the cSMAC and corolla. CD2 binds to lymphocyte-associated antigen 3 (LFA3; dark blue) which is located in the plasma membrane of the antigen-presenting cell (light blue). Among other molecules, TCR/pMHC and CD28/CD80/86 complexes also locate to the cSMAC. LFA-1/ICAM-1 complexes predominantly locate to the pSMAC. See main text for references.

CD2 is an important component in assembly of the IS upon T cell-APC conjugation (19, 32). The IS forms in the contact zone between T cells and APCs upon stable cell-cell interaction and consists of supramolecular activation clusters (SMACs). As shown in Figure 3, the central SMAC (cSMAC) is surrounded by the peripheral SMAC (pSMAC), which in turn is encircled by the distal SMAC (dSMAC). Together, they form a *bull's eye pattern* at the interface between T cells and APCs (76). CD2 is enriched in the IS upon T cell-APC conjugation (77) and appears to play an important role for IS organization (78). It is currently unclear how CD2 affects the location of other membrane molecules in the IS. This effect may be indirectly mediated via the influence of CD2 on actin cytoskeleton rearrangement. IS formation enables accumulation of agonistic signaling molecules at the T-cell-APC interface while excluding membrane molecules that downregulate T cell activation signaling, e.g., CD45, from the center of the IS (32, 78, 79). The relatively short distance between T cells and APCs created by CD2-LFA3 interaction, along with other costimulatory molecules, forms the basis of the kinetic segregation theory (32). This theory describes a model of T cell activation whereby the close contacts formed between T cells and APCs sterically exclude membrane-bound phosphatases with large ECDs (e.g., CD45) from cSMAC and pSMAC. Thus, phosphatases, which might otherwise counteract the relatively high baseline activity of intracellular kinases involved in immunoreceptor tyrosine-based activation motif (ITAM) phosphorylation are sterically excluded from the IS. Consequently, phosphorylation of ITAM domains on the intracellular side of the T cell membrane crosses a threshold which results in T cell activation. For proper IS formation both co-stimulation and specific TCR-MHC binding are required. Some CD2/LFA3 complexes locate to cSMAC, together with other molecules such as CD28/CD80/86 and T cell receptor/peptide MHC (TCR/pMHC) complexes (80). Further, preliminary evidence suggests that clusters of CD2/LFA3 complexes form a ring-like structure between dSMAC and pSMAC termed “corolla” (81).

It has been observed repeatedly that CD2, along with other molecules of the T cell signaling machinery, organizes into microdomains in the IS (28, 82). Upon complete IS formation and given the presence of LFA3, CD2 microclusters tend to reside in the periphery of the IS and it may be speculated that this clustering results from a combination of CD2 translocating to lipid rafts and clustering of CD2 molecules via the ECD of CD2 upon CD2R exposure. As mentioned above, upon cell activation a fraction of CD2 transitions to lipid rafts which are enriched in src family kinases, LAT and components of the T cell signaling machinery but do not contain proteins that connect CD2 and the actin cytoskeleton. It has been shown that clustering of CD2 in the T cell membrane can occur in the absence of the ICD of CD2 (28), possibly mediated by the CD2R epitope (27). However, preliminary evidence indicates that expression of the cytoplasmic tail of CD2 is required for corolla formation (81). A potential explanation for this phenomenon might be that while components of the IS are usually pulled toward cSMAC via centripetal actin-mediated pulling forces, clustered CD2 in lipid rafts may resist this pull more effectively than other IS components as it is not extensively cross-linked with the actin cytoskeleton. This is confirmed by reports that association of CD2 with lck and fyn is required for translocation of CD2 to lipid rafts upon activation (55). This hypothesis would agree with observations that new TCR/CD3 complexes entering the IS emanate from CD2 microdomains in the corolla in the periphery of the IS (81). Following this path of reasoning, CD2 not translocating to lipid rafts would be more extensively linked to the actin cytoskeleton and get pulled into cSMAC. Future research should aim to elucidate what mechanisms underlie the binary positioning of CD2 to the corolla or cSMAC.

## The Role of CD2 in Thymocyte Development

Even before the function or ligand of CD2 was known, its upregulation during the early stages of thymic selection suggested a role in T cell development (83–85). Antibodies to either CD2 or LFA-3 inhibited the binding of thymocytes to thymic epithelial cells (16, 86), revealing involvement of this interaction during thymocyte ontogeny. Furthermore, characterizations of mouse fetal thymocytes reported initial expression of CD2 around day 14 of gestation, followed by an increase to adult levels by day 19 (87–90), whereas CD2 expression in humans has been observed at 9.5 week of gestation (14, 91). Early studies in the mouse thymus demonstrated a correlation between CD2 expression and thymocyte maturation. Namely, CD4^−^ CD8^−^ double-negative (DN) thymocytes expressed low levels, more differentiated CD4^+^ CD8^+^ double-positive (DP) thymocytes intermediate levels, whereas the majority of mature CD4– or CD8-single positive (SP) thymocytes expressed high levels of CD2 (92). Later studies confirmed that CD2 is expressed during late stages of DN maturation into DP thymocytes and maintained on all thymocytes at subsequent developmental stages and ongoing thymic selection events (93). Interestingly, during this pathway into TCR alpha beta T cells, CD2 expression is closely correlated with rearrangement of the TCR beta chain (93), with reports that TCR beta rearrangement stimulates normal CD2 surface expression in developmentally arrested DN thymocytes (94).

However, early studies utilizing CD2 knockout mouse models, or treatment with antibodies against murine CD2, observed normal immunological phenotypes (87, 89, 95), and undermined the view that CD2 plays a major role in T cell development. Although CD2 is not required for the positive selection of T cells, subsequent studies have revealed subtle defects in thymocyte differentiation in mutant mice. CD2-deficient mice displayed altered TCR repertoire selection by affecting Vα gene segment usage in mature T lymphocytes (96). Thus, CD2 can decide the outcome of thymic selection by likely modulating TCR/CD3 stimulation. Notably, tracking CD2-deficient TCR transgenic T cells revealed differential effects of CD2 on thymic selection predicated on the selecting ligand (96–98). On the one hand, CD2-deficiency in T cells bearing TCR with lower affinity for selecting ligand increased the efficiency of positive selection (97). On the other hand, positive selection in TCR transgenic mice with higher affinity for selecting ligand was not perturbed, despite increases in the activation state of DP progenitors in CD2-deficiency (97). Thus, while T cell development was not disengaged in either model, the effect of CD2 on selection was highly variable. Later studies utilizing separate TCR transgenic mouse models confirmed a role for CD2 in thymic selection (96, 98). Specifically, CD2-deficiency in class I-restricted TCR transgene resulted in a differentiation defect during the transition from CD25+ to CD25^−^ CD44^−^ DN immature thymocyte akin the ones observed in mice which lack critical components of pre-TCR signaling (99, 100). Similar experiments in class II-restricted TCR transgenic mice observed a decrease in DP and CD4+ SP thymocytes on a CD2-deficient background (98).

Overall, it is likely that CD2 plays a role at multiple stages of thymic development of T lymphocytes. In addition, these effects of CD2 seems to be dependent upon the associated TCR. While most studies utilizing a variety of CD2-deficient mouse models reported a reduction in the frequency of DP thymocytes (96–98, 101), the observations in regards to thymus cellularity or proportion of other immature thymocyte populations are less consistent. The exact mechanisms of how CD2 functions during thymic development are yet to be determined. One interpretation is that CD2 supports the weak affinity of the pre-TCR signaling in DN cells, by increasing thymocyte to epithelial cell adhesion and/or directly affecting intracellular signaling. Experiments utilizing human CD2 transgenic mice, suggest a key role for the cytoplasmic tail in exerting effects during thymocyte maturation (101). Furthermore, CD2 might affect TCR to peptide-MHC affinity during positive and negative selection allowing immature thymocytes bearing TCRs with relatively high affinities for pMHCs to escape negative selection when CD2 is absent/blocked. The results of increased positive selection upon CD2-deletion for a low-affinity model TCR are surprising, given the opposite role of CD2 in the periphery, however independent studies have made similar findings for other costimulatory molecules such as CD5 and CD28 (102, 103). Investigating possible differences in CD2 cross-talk with the (pre-)TCR complex, in mature T lymphocytes compared to developing thymocytes, might help to elucidate the role of CD2 further. Additionally, re-examining CD2-deficient mouse models through the lens of modern transcriptomics methods that can generate gene expression trajectories paired with single-cell resolution TCR sequencing will provide a more complete view of how CD2 affects T cell differentiation and repertoire generation (104).

## The Role of CD2 in T Cell Activation

T cells are highly motile and scan their environment with the aim of specific antigen recognition, a process involving remodeling of the actin cytoskeleton (105). CD2 has been shown to be enriched in the uropod of scanning T cells along with TCR/CD3 and lipid rafts (106). This indicates an important role of CD2 in APC scanning by T cells prior to IS formation and T cell activation. While the mechanism is yet to be elucidated, CD2 may facilitate formation of the “pre-IS” during probing of APCs by T cells through its influence on T cell signaling or actin cytoskeleton rearrangement. This may reduce the minimum required affinity of TCR/CD3 for pMHC to induce stable cell-cell conjugation and IS formation. A strain of HCMV has been shown to downregulate cell surface expression of LFA3 in host cells and thereby evade CTL cytotoxicity (107). The fact that a pathogen seems to have evolved to evade the CD2/LFA3 pathway underlines importance of this costimulatory pathway in human immunity.

The function of CD2 during activation of naïve T cells has been studied *in vivo* using anti-CD2 antibodies (108, 109), as well as CD2-deficient mouse models (96–98). Anti-CD2 antibody treatment during priming resulted in a reduced T cell response (108), while also protecting T cells from apoptosis upon stimulation by an overly strong TCR signal (109). Interestingly, administration of a single dose of anti-CD2 monoclonal antibody resulted in a sustained hyporesponsiveness of T cells for up to 4 weeks (108), however the mechanism behind this finding has not been elucidated. In addition, naïve CD2-deficient TCR transgenic T cells displayed diminished activation, proliferation, and IFN-γ production upon priming (96–98), however the magnitude of the effect of CD2-deficincy was dependent on the TCR signal intensity. Specifically, T cells expressing TCRs that bind antigen ligands with high affinity are less reliant on CD2 signaling to mount a full immune response. Thus, during primary T cell-mediated immune responses, CD2 is a positive regulator of TCR signaling intensity, and could be crucial in regulating responsiveness to weaker TCR agonists.

The expression of CD2 on memory T cells is higher than on naïve T cells in humans (7, 81, 110) and non-human primates (111, 112). Moreover, CD2 expression is upregulated on activated T cells relative to resting T cells (7, 39). While most costimulatory pathways seem to lose effectiveness in memory and exhausted T cells, CD2-LFA3 retains its ability to contribute to T cell activation (113). Additionally, CD2/LFA3 costimulation seems to play an important role in the reversal of T cell anergy. Whereas, T cells can lose expression of T11.3 upon anergy induction, they regain T11.3 expression only upon extended activation with IL-2 (3). This suggests a role of CD2/LFA3 costimulation in memory T cell activation. Nonetheless, CD2^−^ murine models have shown that CD2 does not seem to be necessary for generation, activation and maintenance of memory T cells (114).

Like naïve T cells, Tregs tend to express lower levels of CD2 when compared to memory T cells in both humans (110) and non-human primates (112). A notable exception are activated Tregs, which display similar CD2 expression to memory T cells (110). The lower expression of CD2 suggests a decreased importance of CD2/LFA3 costimulation in Tregs relative to other T cell subpopulations. Lastly, binding of CD2 by some monoclonal antibodies has been reported to induce apoptosis, suggesting a potential role of CD2 in T cell survival (115, 116). The importance of CD2/LFA3 costimulation for the survival and activation of Tregs and other T cell subpopulations should be investigated more closely.

Recent *in vitro* evidence has shown that CD2/LFA3 costimulation plays an important role in the differentiation of human T cells co-cultured with activated keratinocytes. CD2/LFA3 CoB reduced Signal transducer and activator of transcription 1 (STAT1) signaling and interferon γ production in activated T cells leading to reduced differentiation to a T helper 1 (Th1) phenotype (117, 118). In contrast, differentiation to the Th17 phenotype following stimulation with activated keratinocytes was not affected. Notably, activated keratinocytes expressed LFA3 but did not express B7, while T cells found in psoriatic lesions expressed CD2 but not CD28 (118). These findings indicate that CD2/LFA3 costimulation has an important role in the generation of Th1 cells following T cell activation by non-professional APCs in the epidermis. Potential differences in the role of CD2/LFA3 costimulation in the activation of peripheral and tissue-resident T cells should be investigated further.

## The Role of CD2 in NK Cell Activation

Besides having an important role in NK cell conjugation to target cells (119), CD2 has been shown to recruit CD16 to the NK cell immunological synapse (NKIS) in spontaneous (antibody-independent) NK cell cytotoxicity. CD16 (also known as Fc γ receptor III; FcγRIII) is well-known for its central role in antibody-mediated NK cell cytotoxicity (120), however it still co-localizes to the NKIS with CD2 during spontaneous NK cytotoxicity (121, 122). Similar to what has been observed in T cells, accumulation of CD2 in the NKIS is facilitated by actin cytoskeleton rearrangement and CD2 has a predominantly peripheral positioning in the NKIS (121). NK cells of patients homozygous for a mutation of L66 in CD16 displayed normal ADCC activity but impaired spontaneous cytotoxicity (122, 123). This seemed to derive from an interaction between CD2 and CD16 that leads both to co-localize to the NKIS. L66 in CD16 is important for this interaction and mutation of L66 abrogated interaction between CD2 and CD16. Cells expressing L66H CD16 also display lower CD2 expression than NK cells expressing wild-type CD16 (122). This interaction likely serves to increase the accumulation of activating membrane molecules in the NKIS and thus cross the threshold required for NK cell activation. Given that L66 is situated in the extracellular domain of CD16, CD2, and CD16 may interact via their extracellular domains (122). It may be the case that CD2 serves as a connector between CD16 and the actin cytoskeleton, facilitating repositioning to the NKIS. Thus, similar to its role in T cell-APC conjugation, CD2 has not only an adhesive function on NK cells but also actively recruits activating membrane molecules to the IS, which lowers the threshold for activation.

NKG2C is an activating NK cell receptor which reacts with human cytomegalovirus [HCMV; (124, 125)]. Liu et al. (126) found evidence that the adaptive NK cell response in seropositive HCMV patients involves the synergistic interaction between CD2 and CD16 as well as CD2 and NKG2C. Conversely, no synergistic interaction was observed in HCMV seronegative patients. CD2 ligation with CD16 raised IFN-γ and TNF production, increased activating signaling through CD16 and enhanced antibody-dependent responses (126). These results point toward an important role of CD2 in the functional response of adaptive NK cells. It is especially notable that interaction of CD2 and CD16 enhanced antibody-mediated responses in adaptive NK cells as this was not seen in conventional NK cells as described in the previous paragraph. Similar to T cells, the importance of CD2 may be increased in memory responses of the NK cell compartment (126, 127). Lastly, CD2 seems to synergize with NKG2D in spontaneous cytotoxicity of human NK cells against xenogeneic cells (128) and play an important role in nanotube formation between NK cells and target cell (129).

The general function of CD2 in NK cells and T cells seem similar, however the published literature on the function of CD2 in NK cells to date is limited. Future research should investigate the function of CD2 in NK cell activation more closely to determine similarities and differences in the function of CD2 in NK cells and T cells. Differences in the function of CD2 in T and NK cells would be especially important to consider for therapeutic applications.

## Therapeutic Applications of CD2-LFA3 Costimulatory Blockade

Organ transplantation and certain types of autoimmune disorders, conditions characterized by excessive activation of T cells and NK cells, constitute the natural target for CD2-LFA3 CoB. In organ transplantation, CD2-LFA3 CoB could e.g., be used in induction therapy and maintenance immunosuppression to prolong graft survival and minimize the risk for allograft rejection. A substantial part of CD2-LFA3 CoB clinical studies have been conducted using depleting anti-CD2 mAbs which provides two MoAs: Depletion of CD2-expressing cells and CD2/LFA3 costimulatory blockade. There exists little evidence on direct immune modulation via CD2/LFA3 CoB *in vivo*. Research on non-depleting anti-CD2 agents should be conducted to elucidate the contribution of each MoA to the effects seen with depleting anti-CD2 agents.

Chavin et al. (130) showed that treatment with anti-CD2 mAb prolonged xenograft islet survival and cardiac allograft survival in mice. More recently, it was shown that treatment of transgenic mice expressing human CD2 in a transfer colitis model with anti-human CD2 mAb CB.219 reduced intestinal inflammation and prolonged survival (131). Interestingly, efficacy varied between different anti-CD2 mAbs with two other anti-human CD2 mAbs (Clones 35.1 or 8E5, respectively) showing markedly lower or no efficacy, depending on the assessed parameter. These findings suggest that differences in binding properties (i.e., epitope or affinity) may potentially influence the effect of CD2-LFA3 CoB. Further, addition of CD2-LFA3 CoB via the fusion protein Alefacept to a CoB-based regimen extended allograft survival in rhesus macaques and depleted peripheral effector memory T cells (111). It is difficult to deduce how much of the observed effects in these studies stem from CD2-LFA3 CoB and how much from depletion of CD2^+^ cells. In this regard, it should be noted that while anti-CD2 mAbs induced strong depletion of peripheral T cells (siplizumab or BTI-322 [the original rat IgG2b version of siplizumab]) in chimpanzees, only moderate T cell depletion was seen in secondary lymphoid tissue (132). Given incomplete depletion in secondary lymphoid tissue and assuming sufficient tissue distribution, CD2-LFA3 CoB could become relatively more important as an MoA of CD2-targeting biologics in lymph nodes and other tissues where depletion of CD2^+^ cells is milder than in peripheral blood. Notably, depletory anti-CD2 mAb treatment induced a relative enrichment of Tregs in cynomolgus macaques (112) and in mixed lymphocyte reactions using human peripheral blood mononuclear cells (110). The study conducted by Podesta et al. (110) is especially notable since it reported an enrichment of CD45RA^−^ Tregs using a depletory anti-CD2 mAb. CD2 expression levels on CD45RA^−^ Tregs tends to be similar to the expression level seen on memory T cells. These results indicate that there may be other mechanisms than depletion which contribute to Treg enrichment with anti-CD2 mAbs. The approach of combining depletion of disease-mediating cells and CoB of remaining or newly maturing T and NK cells opens an alternative view in the design of treatment strategies for pathologies characterized by an uncontrolled immune response.

Most clinical studies investigating CD2-LFA3 CoB have been conducted using two biologic drugs: (1) Alefacept, a fusion protein of the ECD of LFA3 and an IgG1 Fc-fragment; and (2) siplizumab (previously known as MEDI-507), a humanized anti-CD2 monoclonal IgG1 antibody. Alefacept has been clinically studied for treatment of psoriasis (133) and new-onset type I diabetes (134) and for use in transplant induction therapy (135, 136). Alefacept did not seem to elicit notable additional adverse events compared to control groups and induced selective (albeit modest) depletion of peripheral memory T cells. Similarly, siplizumab has been investigated for treatment of plaque psoriasis (137), transplant induction therapy (138, 139), stem cell transplantation (140), graft-vs.-host disease (141) and T cell malignancies (142). In addition, siplizumab has been used in a treatment protocol aimed at inducing renal allograft tolerance, in which kidney transplantation is augmented by donor bone marrow infusion, in order to induce transient mixed chimerism (143, 144). Donor-specific Tregs were found to be enriched in this cohort, possibly due to selective depletion by siplizumab (145, 146). The results of these studies have stimulated further development of siplizumab, in order to resume clinical efforts toward establishment of allograft tolerance and treatment of autoimmune conditions. Collectively, reported clinical data on anti-CD2 agents have shown that this is a safe treatment modality that can be used in a broad spectrum of patients. Interestingly, the CD2 activation epitope CD2R has been reported to be upregulated on T cells in the synovial fluid of rheumatoid arthritis (RA) patients and in the peripheral blood of patients with juvenile RA, systemic lupus erythematosus, ankylosing spondylitis, and Lyme disease (147). Thus, therapeutic anti-CD2R/T11.3 mAbs could potentially be used to more specifically target autoreactive T cells in these conditions. Lastly, Tregs isolated from the peripheral blood of multiple sclerosis patients tended to exhibit reduced suppressive function relative to Tregs isolated from heathy controls (148). CD2/LFA3 CoB may be able to augment peripheral Treg-mediated suppression of autoreactive T cells in these patients and thus promote peripheral tolerance. Clinical trials in the discussed autoimmune conditions are needed to evaluate the efficacy of CD2-targeting therapies in these patient groups.

To date, most clinical trials of CD2-LFA3 CoB have focused on peripheral T (and NK) cells, with limited attention being paid to depletion and/or CD2/LFA3 CoB of tissue-resident T and NK cells. Data about tissue-resident NK cells, e.g., in lymph nodes, and their response to CD2-LFA3 CoB may be especially interesting as the majority of lymph node NK cells have a CD56^bright^ phenotype (149) which has been shown to express CD2 at markedly higher levels than CD56^dim^ NK cells (150), the most common phenotype in peripheral blood. NK cell count in peripheral blood seems decreased in many autoimmune conditions. However, there seems to be a concurrent enrichment of NK cells in tissues affected by autoimmune disease, suggesting an increased migration from peripheral blood to tissue (151). CD56^bright^ NK cells have been shown to have relatively little cytotoxic capacity but a relatively high cytokine secretion potential (149). CD2-LFA3 CoB treatments enabling more selective depletion and inhibition of T (and NK) cells could be desirable in a variety of clinical settings. Commercially available depletory agents such as anti-thymocyte globulin (ATG) and Alemtuzumab bind other cell types in addition to T and NK cells and accordingly do not allow for selective depletion or inhibition of T and NK cells. Use of CD2 as a target for depletion and/or CoB may enable a reduction in off-target effects compared to currently available depletory agents. Inhibition of spontaneous NK cell cytotoxicity by blocking the association of CD2 and CD16 and interfering with NK-target cell conjugation via the CD2-LFA3 pathway through anti-CD2 mAbs could be used in prevention or treatment of transplant rejection as well for treatment of autoimmune conditions deriving in part from spontaneous NK cell cytotoxicity. Effectiveness of inhibiting NK cell activation via CD2-LFA3 CoB in these conditions likely depends on the mode of NK cell activation inherent to the disease. If NK cell activation is triggered via autoantibodies the potential of CD2-LFA3 CoB is likely low because CD2-LFA3 co-stimulation does not seem important for ADCC (122). However, if NK cell activation is spontaneous CD2-LFA3 CoB may have an inhibitory effect, especially if association of CD2 and CD16 is prevented in addition to CD2-LFA3 binding.

Patients with CD2^+^ lymphomas may benefit from depletory anti-CD2 treatments. Administration of siplizumab to NOD/SCID mice inoculated with adult T cell leukemia prolonged survival given expression of intact Fcγ receptors (152). A phase I study of siplizumab added to chemotherapy for treatment of patients with peripheral T cell lymphoma resulted in 84.5% overall response rate (OR), with 62% of patients achieving complete remission (CR). However, long-term progression-free survival was not improved (142) and there was no significant increase in observed OR and CR rates over other available treatments of lymphoma patients (153). An alternative to anti-CD2 mAbs, CD2-targeting chimeric antigen receptor T cells could be employed for treatment of CD2^+^ lymphomas. Indeed, there exist efforts to engineer CART cell treatments against T cell antigens like CD2 or CD7, which are overexpressed in T-cell acute lymphoblastic leukemia. To maximize anti-tumor activity, the target antigen in these CART cells is deleted (154). Moreover, due to the broad expression of LFA3 on CD2- lymphomas and the important role of CD2 in T cell costimulation, investigators have highlighted the benefit of using CD2 costimulation on CART cells (155). Notably, even if the activation profile of CD2 is not desired, the extracellular and transmembrane domains of CD2 can be fused with intracellular domains of other stimulating cell surface antigens. This approach combines enhanced target cell-binding by CART cells with desired activation signaling to maximize therapeutic efficacy (156, 157). Other conditions exist which could potentially be treated with CD2^+^ cell depletion or CD2-LFA3 CoB, e.g., NK-type lymphoproliferative disease of granular lymphocytes (158) or TAP deficiency (159).

## Conclusion

Although CD2 has traditionally been considered a non-essential adhesion molecule, evidence that has emerged since the last comprehensive review by Davis and van der Merwe (19), confirms their hypotheses about the multifunctionality of CD2 and its important roles in cell-cell adhesion, IS formation, IS architecture, IS composition and recruitment of intracellular kinases to the IS. Due to the increased expression of CD2 on activated and memory T cells as well as its importance for spontaneous NK cell cytotoxicity, CD2-targeting therapies could become a potent tool for modulating the activation of these cell types in transplant patients or individuals suffering from autoimmune disease. Thus, it seems likely that the recent renewal of interest in CD2 immunology that we have examined in this review will have important clinical applications in the near future.

## Author Contributions

CB and FC analyzed the literature and wrote the first manuscript. All authors were involved in reviewing and editing the manuscript.

## Conflict of Interest

CB, FC, FS, SB, DS, EB, and DB are employees of and/or consultants to ITB-MED. SB, DS, EB, and DB own shares in ITB-MED. HP is an intern at ITB-MED.

## Abbreviations

ADCC: Antibody-dependent cell cytotoxicity
APC: Antigen-presenting cell
CD: Cluster of differentiation
CD2AP: CD2-associated protein
CD2BP: CD2-binding protein
CD2R: CD2 restricted epitope
CIN85: Cbl-interacting protein of 85 kDa
CMS: Cas ligand with multiple SH3 domains
CoB: Costimulatory blockade
cSMAC: central supramolecular activation motif
CTL: Cytotoxic T lymphocyte
DN: CD4^−^ CD8^−^ double-negative
DP: CD4^+^ CD8^+^ double-positive
Dok: Docking protein
dSMAC: distal SMAC
ECD: Extracellular domain
FAK: Focal adhesion kinase Fcγ: Fragment crystallizable gamma
FcγR: Fragment crystallizable gamma receptor
GYF: glycine tyrosine phenylalanine
HCMV: Human cytomegalovirus
ICD: Intracellular domain
IS: Immunological synapse
ITAM: Immunoreceptor tyrosine-based activation motif
JNK: c-Jun N-terminal kinase LAT: Linker for activation of T cells
Lck: Lymphocyte-specific Protein Tyrosine Kinase
mAb: Monoclonal antibody
MAPK: Mitogen-activated protein kinase
MTOC: Microtubule-organizing center
mTOR: Mammalian target of rapamycin NCAM: Neural cell adhesion molecule
NFAT: Nuclear factor of activated T-cells
NF-κB: Nuclear factor κB
NKIS: NK cell immunological synapse
NOD/SCID: Non-obese diabetic/severe combined immunodeficiency
PDB: Protein data bank
PI3K: Phosphatidylinositol 3-kinase
PKCθ: Protein Kinase C θ
PLCγ1: Phospholipase C γ1
pMHC: peptide Major histocompatibility complex
pSMAC: peripheral SMAC
Pyk2: Protein Tyrosine Kinase 2 Beta
RA: Rheumatoid arthritis
SH3: Src homology 3
SLP76: SH2 Domain-Containing Leukocyte Protein of 76 KDa
STAT1: Signal transducer and activator of transcription 1
T1DM: Type I diabetes mellitus
TCR: T cell receptor
Th1: T helper 1
Th17: T helper 17
Vav1: Vav Guanine Nucleotide Exchange Factor 1
WASp: Wiskott-Aldrich Syndrome Protein
ZAP70: Zeta-Chain Associated Protein Kinase of 70 kDa.
